# Adherence to Follow-Up Colonoscopy After a Positive Stool-Based Test in Patients Aged 45–49 Years: Real-World Differences Between Multitarget Stool DNA Testing and Fecal Immunochemical or Fecal Occult Blood Testing

**DOI:** 10.1016/j.gastha.2025.100706

**Published:** 2025-05-16

**Authors:** Mallik Greene, Brad Stieber, A. Burak Ozbay, Derek Ebner, A. Mark Fendrick, Jordan J. Karlitz

**Affiliations:** 1 Exact Sciences Corporation, Madison, Wisconsin; 2 Mayo Clinic, Rochester, Minnesota; 3 Division of General Medicine, Department of Internal Medicine, University of Michigan, Ann Arbor, Michigan

**Keywords:** Adherence, Colonoscopy, Colorectal Cancer, Disparities, Mt-sDNA, Stool-Based Tests

## Abstract

**Background and Aims:**

A positive stool-based test (SBT) result requires timely follow-up colonoscopy (FU-CY) to realize the full benefits of screening recommendations. The current investigation examined the real-world rate of, and time to, FU-CY adherence among patients aged 45–49 years who were screened with either a fecal immunochemical test (FIT)/fecal occult blood test (FOBT) or a multitarget stool DNA test (mt-sDNA).

**Methods:**

This retrospective study utilized a large integrated national multipayer claims database. Patients were aged 45–49 years and had a positive SBT result between January 1, 2017, and June 30, 2022 (study period); the first screening test result served as the index date. The primary outcome was adherence to FU-CY, defined as evidence of a colonoscopy (based on Current Procedural Terminology codes) within 12 months of a positive SBT.

**Results:**

A total of 13,921 patients with a positive SBT result were identified during the study period (FIT/FOBT, n = 6429; mt-sDNA, n = 7492). Among those with a positive SBT, FU-CY adherence was significantly higher among patients screened with mt-sDNA compared to those with FIT/FOBT (85.0% vs 35.2%, respectively; *P* < .01). Within the first 90 days, 62.9% of mt-sDNA users completed FU-CY, compared to only 22.8% of FIT/FOBT users. The mean time to FU-CY was 74.5 days for those screened with mt-sDNA (median, 53 days), compared to 90.8 days for FIT/FOBT (median, 63 days).

**Conclusion:**

FU-CY adherence rates were significantly higher in patients screened with mt-sDNA compared to those screened with FIT/FOBT. These results suggest that in younger patient populations, mt-sDNA may be a promising tool to improve FU-CY adherence rates and enhance the quality of colorectal cancer screening.

## Introduction

Colorectal cancer (CRC) remains a major contributor to cancer-related mortality in the United States, despite being one of the most preventable cancers or malignancies.^1^ CRC ranks as the leading cause of cancer among men under 50 and the second leading cause among women in the same age group.^1^ Early-onset CRC, defined as diagnosis before age 50, accounts for approximately 10% of all initial CRC cases ^1^^,^^2^ and has been increasing annually by 1%–2% since the mid-1990s, in contrast to declining rates observed among older adults.^3^, ^4^, ^5^, ^6^ In addition, the rate of more advanced-stage CRC in adults aged 45–49 has increased relative to other age groups, highlighting the critical need for screening adherence.^7^ Globally, early-onset CRC is rising,^5^^,^^8^, ^9^, ^10^ and US projections estimate that rates could double by 2030, potentially impacting over 27,000 individuals under age 50.^11^

In response to the rising early-onset CRC rates, the US Preventive Services Task Force,^12^ the American Cancer Society,^13^ the American College of Gastroenterology,^14^ and the US Multi-Society Task Force on Colorectal Cancer ^14^ have recently harmonized their clinical guidelines, lowering the recommended screening age from 50 to 45 years of age in average-risk individuals. These updated recommendations reflect a paradigm shift aimed at promoting earlier screening to detect precursors to cancer, such as precancerous polyps, and early-stage (localized) CRC, as early detection has been shown to significantly improve the likelihood of a favorable prognosis.^15^, ^16^, ^17^, ^18^ Despite updated guidelines, adherence among this population remains suboptimal.^19^, ^20^, ^21^, ^22^ A recent study found that between 2019 and 2021, only about 19.7% of the 19 million eligible adults aged 45–49 years were current with CRC screening.^23^ This gap in screening is particularly concerning given the difference in outcomes between early- and late-stage diagnosis. Research indicates that when CRC is identified at a localized stage, it has a promising 5-year survival rate of up to 90%.^24^, ^25^, ^26^ In contrast, survival rates drastically decrease to between 12% and 14% once the disease progresses to metastatic stages.^25^^,^^27^

Noninvasive, at-home screening options for CRC currently include stool-based tests (SBTs) such as fecal immunochemical tests (FITs), fecal occult blood tests (FOBTs), and multitarget stool DNA tests (mt-sDNA; Cologuard, a product of Exact Sciences Laboratories LLC [ESL]).^13^^,^^28^^,^^29^ While SBTs offer a more accessible option for CRC screening,^13^^,^^29^, ^30^, ^31^ their efficacy depends on timely follow-up colonoscopy (FU-CY), within 6 to 9 months, for individuals with an abnormal SBT result.^12^^,^^32^, ^33^, ^34^, ^35^, ^36^ The US Multi-Society Task Force on CRC underscores the importance of timely FU-CY, setting a minimum benchmark of at least 80% completion after a positive SBT.^37^ However, this benchmark is often not achieved, with previous studies reporting variable FU-CY rates across different ages within both 6 ^33^^,^^34^^,^^38^, ^39^, ^40^, ^41^ and 12 months.^42^^,^^43^

Although FU-CY adherence has been evaluated broadly, rates among patients aged 45–49 have historically been overlooked due to CRC screening guidelines targeting individuals aged 50 and older. To address this critical gap in the literature, this study evaluated FU-CY adherence rates after a positive SBT among patients under 50 years old. Given the increasing emphasis on earlier CRC screening, this research was guided by two primary objectives: (1) to evaluate real-world FU-CY rates among patients aged 45–49 after a positive SBT (FIT/FOBT or mt-sDNA), and (2) to assess the time interval to FU-CY completion.

## Methods

### Data Source

This retrospective study linked two large databases. The first was the Komodo Research Data (KRD) + MapEnhance Komodo Lab database, a comprehensive and representative administrative claims database that includes closed claims data from over 165 million lives across more than 150 payer sources. The second was the ESL database, which provides complete information on mt-sDNA orders for millions of patients annually, including laboratory test results, patient outreach, health systems, payers, and basic demographic data. Linking the KRD and ESL data created the KRD-ESL dataset, which offers complete information on mt-sDNA and FIT/FOBT testing and results, along with comprehensive demographic and health information from the KRD data. The study was considered exempt research under 45 CFR § 46.104(d)(4) as it involved only the secondary use of data that were deidentified in compliance with the Health Insurance Portability and Accountability Act, specifically, 45 CFR § 164.514.

### Study Design and Sample Selection

Eligible patients were aged 45–49 years and had a positive SBT result between January 1, 2017, and June 30, 2022 (study period); the first screening test result served as the index date. Eligible patients were required to be continuously enrolled in a health plan for a minimum of 6 months prior to the index date and 1 year after (follow-up period) the index date. Patients were excluded if they had a high-risk CRC diagnosis (based on International Classification of Diseases, Tenth Edition codes) within 6 months prior to the index date, missing demographic information, or no evidence of healthcare utilization in the 12 months following the test.

### Variables and Outcomes

The primary outcome was adherence to FU-CY, defined as evidence of a colonoscopy (based on Current Procedural Terminology codes) within 12 months after a positive SBT. In addition, the length of time between a positive SBT and FU-CY was a secondary outcome. Patient demographic characteristics included age group, sex, ethnicity, payer type, geographical region, and stratification by index year.

### Statistical Analyses

We used descriptive statistics to summarize baseline characteristics overall and by payer type, presented as frequencies and percentages. We reported the primary outcome, FU-CY adherence rate, overall and by payer type, along with corresponding descriptive statistics, analysis of variance, and chi-square test results. Multivariable logistic regression analysis examined factors associated with adherence (yes/no); independent variables included stool test type, index year, sex, payer type, race/ethnicity, and geographical region. All statistical analyses were performed using R version 4.2.2 (The R Foundation for Statistical Computing, Vienna, Austria).

## Results

### Patient Characteristics

We identified 13,921 eligible patients aged 45–49 years with positive stool-based screening test results between January 1, 2017, and June 30, 2022 (mt-sDNA, n = 7492; FIT/FOBT, n = 6429). Baseline demographic characteristics stratified by screening modality (mt-sDNA vs FIT/FOBT) are presented in Table 1. Overall, the sample comprised 7182 (51.6%) females and 6739 (48.4%) males, with the majority identifying as White (50.8%), followed by Other (25.2%), Hispanic or Latino (10.2%), Black or African American (9.0%), and Asian or Pacific Islander (4.8%). Most patients (80.3%) had commercial insurance, with a higher proportion among mt-sDNA patients (84.1%) compared to FIT/FOBT patients (75.8%). Regionally, most patients were from the South (40.3%) and Northeast (26.0%), with smaller representation from the Midwest (20.0%) and West (13.7%). Lastly, most mt-sDNA tests were conducted in 2021 (39.2%) and 2022 (50.1%), while FIT/FOBT tests were more evenly distributed across earlier years, with the highest proportions in 2017 (18.9%) and 2018 (25.5%).

**Table 1:** Patient Demographic Characteristics

	Mt-sDNA test (*N* = 7492)	FIT/FOBT test (*N* = 6429)	Overall (*N* = 13,921)
Age, *n* (%)			
45–49	7492 (100.0%)	6429 (100.0%)	13,921 (100.0%)
Sex, *n* (%)			
Female	3728 (49.8%)	3454 (53.7%)	7185 (51.6%)
Male	3764 (50.2%)	2975 (46.3%)	6739 (48.4%)
Race/Ethnicity, *n* (%)			
Asian or Pacific Islander	186 (2.5%)	485 (7.5%)	671 (4.8%)
Black or African American	534 (7.1%)	712 (11.1%)	1246 (9.0%)
Hispanic or Latino	504 (6.7%)	921 (14.3%)	1425 (10.2%)
Other	2995 (40.0%)	513 (8.0%)	3508 (25.2%)
White	3273 (43.7%)	3798 (59.1%)	7071 (50.8%)
Payer type, *n* (%)			
Commercial	6303 (84.1%)	4874 (75.8%)	11,177 (80.3%)
Medicaid	1012 (13.5%)	1271 (19.8%)	2283 (16.4%)
Medicare advantage	143 (1.9%)	238 (3.7%)	381 (2.7%)
Medicare fee-for-service	34 (0.5%)	46 (0.7%)	80 (0.6%)
Geographic region, *n* (%)			
Midwest	1066 (27.4%)	2399 (19.1%)	2786 (20.0%)
Northeast	1892 (48.6%)	6733 (53.6%)	3625 (26.0%)
South	492 (12.6%)	1898 (15.1%)	5607 (40.3%)
West	100 (2.6%)	316 (2.5%)	1903 (13.7%)
Index year, *n* (%)			
2017	8 (0.1%)	1215 (18.9%)	1223 (8.8%)
2018	11 (0.1%)	1638 (25.5%)	1649 (11.8%)
2019	173 (2.3%)	1058 (16.5%)	1231 (8.8%)
2020	609 (8.1%)	807 (12.6%)	1416 (10.2%)
2021	2936 (39.2%)	1111 (17.3%)	4047 (29.1%)
2022	3755 (50.1%)	600 (9.3%)	4355 (31.3%)

### FU-CY Adherence Rates in FIT/FOBT Vs Mt-sDNA-Positive Patients

Disparities in adherence rates for FU-CY within 365 days after a positive stool-based screening test, stratified by subgroup, are presented in Table 2. Overall, adherence to FU-CY was significantly higher with mt-sDNA compared to FIT/FOBT among patients aged 45–49 years (85.0% vs 35.2%; *P* < .01). When stratified by sex, adherence was significantly higher with mt-sDNA compared to FIT/FOBT for both females (84.0% vs 34.3%; *P* < .01) and males (86.0% vs 36.1%; *P* < .01), with slightly higher adherence rates observed among males, regardless of screening modality. Racial and ethnic disparities were also observed: adherence to FU-CY remained significantly higher with mt-SDNA compared to FIT/FOBT across all groups (all *P* < .01). Among Asian or Pacific Islander and White patients, mt-sDNA adherence rates were 86.6% and 85.5%, respectively, compared to 30.5% and 33.5% with FIT/FOBT, respectively. Similar trends were observed among Black or African American (82.8% vs 43.4%), Hispanic or Latino (84.3% vs 36.8%), and patients categorized as Other (84.8% vs 37.4%) (all *P* < .01).

**Table 2:** Comparison of FU-CY Adherence Rates by Screening Modality

	Mt-sDNA test (*N* = 6369)	FIT/FOBT test (*N* = 2261)	*P* value
Age, *n* (95% CI)			
45–49	85.0% (84.2%–85.8%)	35.2% (34.0%–36.3%)	<.01
Sex, *n* (95% CI)			
Female	84.0% (82.8%–85.2%)	34.3% (32.8%–35.9%)	<.01
Male	86.0% (84.9%–87.1%)	36.1% (34.4%–37.9%)	<.01
Race/Ethnicity, *n* (95% CI)			
Asian or Pacific Islander	86.6% (81.7%–91.5%)	30.5% (26.4%–34.6%)	<.01
Black or African American	82.8% (79.6%–86.0%)	43.4% (39.8%–47.0%)	<.01
Hispanic or Latino	84.3% (81.2%–87.5%)	36.8% (33.7%–39.9%)	<.01
Other	84.8% (83.6%–86.1%)	37.4% (33.2%–41.6%)	<.01
White	85.5% (84.3%–86.8%)	33.5% (32.0%–35.0%)	<.01
Payer type, *n* (95% CI)			
Commercial	86.3% (85.5%–87.2%)	33.5% (32.2%–34.8%)	<.01
Medicaid	78.1% (75.5%–80.6%)	39.6% (36.9%–42.3%)	<.01
Medicare advantage	81.1% (74.7%–87.5%)	43.7% (37.4%–50.0%)	<.01
Medicare fee-for-service	67.6% (51.9%–83.4%)	45.7% (31.3%–60.0%)	.051
Geographic region, *n* (95% CI)			
Midwest	86.5% (85.1%–87.9%)	40.8% (36.5%–45.2%)	<.01
Northeast	84.1% (82.1%–86.1%)	32.7% (30.8%–34.6%)	<.01
South	84.0% (82.7%–85.4%)	27.3% (25.6%–29.0%)	<.01
West	85.9% (83.6%–88.3%)	57.2% (54.2%–60.2%)	<.01
Index year, *n* (95% CI)			
2017	50.0% (15.4%–84.6%)	31.6% (29.0%–34.2%)	.27
2018	81.8% (59.0%–104.6%)	23.1% (21.0%–25.1%)	<.01
2019	87.3% (82.3%–92.2%)	33.0% (30.2%–35.8%)	<.01
2020	83.7% (80.8%–86.7%)	36.2% (32.9%–39.5%)	<.01
2021	85.3% (84.0%–86.6%)	45.5% (42.6%–48.5%)	<.01
2022	85.0% (83.8%–86.1%)	58.7% (54.7%–62.6%)	<.01

CI, confidence interval.

For most payer types, adherence to FU-CY was significantly higher with mt-sDNA compared to FIT/FOBT, including among patients with commercial insurance (86.3% vs 33.5%), Medicaid (78.1% vs 39.6%), and Medicare Advantage (81.1% vs 43.7%) (all *P* < .01). Among Medicare fee-for-service patients, adherence was also higher with mt-sDNA (67.6% vs 45.7%), though the difference approached but did not reach statistical significance (*P* = .051). Regionally, adherence to FU-CY was significantly higher with mt-sDNA compared to FIT/FOBT across all geographic regions (all >80%; all *P* < .01), with the highest mt-sDNA adherence rates observed in the Midwest (86.5%), followed by the West (85.9%), Northeast (84.1%), and South (84.0%). In contrast, FIT/FOBT adherence was highest in the West (57.2%), followed by the Midwest (40.8%), Northeast (32.7%), and South (27.3%). Additionally, adherence to FU-CY was significantly higher with mt-sDNA compared to FIT/FOBT across all index years, except for 2017, when no significant difference was observed (*P* = .2652). By 2022, mt-sDNA adherence reached 85.0%, compared to 58.7% for FIT/FOBT (*P* < .001).

### Predictors of FU-CY

Predictors of adherence to FU-CY were assessed using multivariable logistic regression, with results presented in Table 3. Compared with patients screened using FIT/FOBT, those screened with mt-sDNA had 6.7 times higher odds of adhering to FU-CY (*P* < .01). Stratification by insurance type revealed that Medicaid patients had 26% lower odds of adherence compared to those with commercial insurance (*P* < .01), while patients with Medicare Advantage showed no significant difference (*P* = .80).

**Table 3:** Logistic Regression Predictors of FU-CY

Coefficient	Estimate	Odds ratio (95% CI)	*P* value
Intercept	−0.94	0.39 (0.31, 0.50)	<.01
Stool test (reference = FIT-FOBT)			
mt-sDNA	1.90	6.69 (5.96, 7.51)	<.01
Shipment year (reference = 2017)			
2018	−0.39	0.68 (0.57, 0.80)	<.01
2019	0.17	1.19 (1.00, 1.41)	.05
2020	0.33	1.39 (1.17, 1.65)	<.01
2021	0.63	1.88 (1.60, 2.20)	<.01
2022	0.74	2.10 (1.78, 2.49)	<.01
Gender (reference = female)			
Male	0.14	1.15 (1.06, 1.25)	<.01
Payer type (reference = commercial)			
Medicaid	−0.30	0.74 (0.66, 0.83)	<.01
Medicare advantage	0.03	1.03 (0.81, 1.31)	.80
Medicare FFS	−0.27	0.77 (0.46, 1.29)	.32
Race/Ethnicity (reference = Asian or Pacific Islander)			
Black or African American	0.57	1.76 (1.41, 2.20)	<.01
Hispanic or Latino	0.24	1.27 (1.03, 1.57)	.03
Other	0.24	1.27 (1.03, 1.55)	.02
White	0.27	1.32 (1.09, 1.58)	<.01
Geographic region, (reference = Midwest)			
Northeast	−0.20	0.82 (0.72, 0.94)	<.01
South	−0.40	0.67 (0.60, 0.76)	<.01
West	0.57	1.77 (1.51, 2.07)	<.01

CI, confidence interval; FFS, fee-for-service.

Adherence also varied by gender and race/ethnicity, with male patients having 15% higher odds of adherence compared to females (*P* < .01). Compared with Asian or Pacific Islander patients, Black or African American patients had 76% higher odds of adherence (*P* < .01), while Hispanic or Latino (27% higher odds; *P* = .03), White (32% higher odds; *P* < .01), and individuals categorized as “Other” (27% higher odds; *P* = .02) also demonstrated higher adherence. Lastly, regional differences were observed, with patients in the South having 33% lower odds of adherence (*P* < .01), and those in the Northeast having 18% lower odds (*P* < .01), compared to patients in the Midwest.

### Rates of FU-CY Over Time

**Figure 1:**
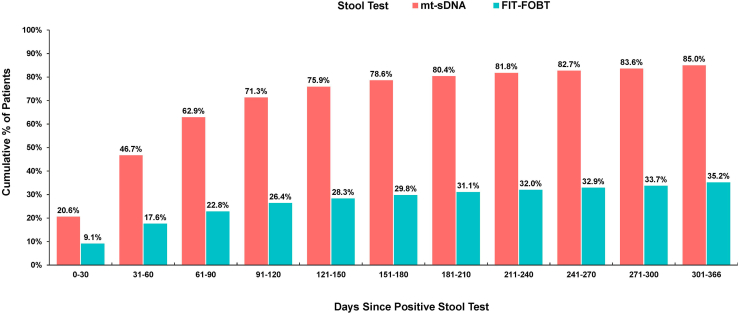
Cumulative adherence to FU-CY within one year after a positive stool-based test.

Across all patients, 85.0% of those with a positive mt-SDNA test underwent a FU-CY within 1 year, compared to only 35.2% of those with a positive FIT/FOBT test (Figure 1). Among patients who completed a FU-CY within 1 year, the mean time to FU-CY was shorter in the mt-sDNA cohort (74.5 days; median, 53 days) compared to the FIT/FOBT cohort (90.8 days; median, 63 days). A higher proportion of patients with a positive mt-sDNA result completed a timely FU-CY within 180 days compared to those with a positive FIT/FOBT result (78.6% vs 29.8%; *P* < .01), suggesting that approximately 92% (78.6/85.0 × 100) of FU-CYs completed within 12 months were already completed by 180 days.

## Discussion

This retrospective study is one of the first to examine real-world differences between the mt-sDNA test and FIT/FOBT in average-risk participants aged 45–49 years. The overall adherence to FU-CY within 1 year of a positive test result was significantly higher among mt-sDNA users (85.0%) compared to FIT/FOBT users (35.2%). Notably, the adherence rate after a positive mt-sDNA test exceeded the 80% FU-CY benchmark for screening completion after a positive SBT.^37^ Subgroup analyses stratified by gender, race/ethnicity, region, index year, and payer type revealed that FU-CY adherence among mt-sDNA users ranged from 67.6% to 87.3%, which was significantly greater than among FIT/FOBT users, whose adherence ranged from 23.1% to 58.7%. Across payer types, adherence was higher among patients screened with an mt-sDNA test. Even among patients with commercial insurance, who potentially face fewer barriers to completing a FU-CY regardless of the initial screening modality compared to those with other insurance types, adherence was 86.3% with mt-sDNA compared to 33.5% for FIT-FOBT.

Previous research has shown variability in FU-CY rates after a positive SBT, although evidence comparing the follow-up adherence of mt-sDNA versus FIT/FOBT among adults aged 45–49 remains limited. A prior retrospective study of commercially insured and Medicare Advantage enrollees in the United States found that only 65% of patients with a positive SBT result completed a FU-CY within 6 months, with substantial variation by test type: 46.0% after a positive FIT and 72.1% after a positive mt-sDNA.^44^ Although this study specifically targeted older adults aged 50–75, it provides a useful barometer for understanding FU-CY adherence and the variability associated with different SBTs. Another retrospective cohort study examined FU-CY adherence among individuals aged 40 and older with positive FIT or mt-sDNA results.^38^ In that study, FU-CY completion within 6 months was significantly higher among mt-sDNA users compared to FIT users (71.5% vs 46.7%). However, both studies reported FU-CY rates below the 80% benchmark and notably lower than the rates observed in our study, particularly among mt-sDNA users. The high FU-CY rate with mt-sDNA (78.6%) after 6 months reported in the current study is especially important, given that delays of more than 6 months, compared to follow-up within 1 month after a positive FIT test, are associated with an increased risk of CRC and advanced-stage CRC.^45^

Predictors of adherence to FU-CY, assessed using multivariable logistic regression, highlighted several key factors influencing patient behavior in our study. Patients screened with mt-sDNA tests had 569% greater odds of adherence to FU-CY compared to those screened with FIT/FOBT. We also observed adherence patterns that evolved over time. Compared to 2017, the odds of adherence decreased by 32% in 2018 but improved steadily in the following years. By 2019, the odds of adherence increased slightly, rising by 19% compared to 2017. This upward trend continued to be observed, with odds of adherence increasing 39% in 2020, 88% in 2021, and 110% in 2022. This improvement may reflect growing awareness of CRC screening guidelines, updated in 2021, along with enhancements in outreach efforts and broader adoption of mt-sDNA testing during the study period. Stratification by insurance type revealed that Medicaid patients had 26% lower odds of adherence compared to those with commercial insurance, while those with Medicare Advantage showed no significant difference. Additional research is needed to better understand the differences in FU-CY adherence associated with payer type. One possible explanation is that systemic barriers exist that limit access to FU-CY for Medicaid patients. For instance, a recent study found that many gastroenterologists were unaware of the Medicaid expansion policies covering colonoscopies, which may limit the number of Medicaid patients they see in their practices, as they cite issues with lower reimbursement and “patient compliance.”^46^

Adherence also varied by demographics, with male patients having 15% higher odds of adherence than females, a finding that may warrant further investigation into gender-specific barriers, such as differences in health-seeking behavior. Racial and ethnic disparities were also observed, with Black or African American patients having 76% greater odds of adherence compared to Asian or Pacific Islander patients. Historically, Black Americans have had the lowest CRC screening rates and tend to present with CRC at earlier ages, often with more insidious onset and higher risk of morbidity compared to Caucasians.^47^^,^^48^ However, the higher adherence observed in this study among Black or African American patients, compared to the reference group (Asian or Pacific Islander), highlights a significant opportunity to address longstanding screening disparities and improve outcomes for Black Americans through targeted interventions. Hispanic or Latino patients (27% higher odds), White patients (32% higher odds), and individuals categorized as “Other” (27% higher odds) also demonstrated higher adherence. Geographic disparities revealed that patients in the South had 33% lower odds of adherence, while those in the Northeast had 18% lower odds compared to patients in the Midwest.

Finally, our study showed a significant variation in cumulative adherence to FU-CY within 1 year after a positive SBT. The mean time to FU-CY was shorter in the mt-sDNA cohort (74.5 days; median, 53 days) compared to the FIT/FOBT cohort (90.8 days; median, 63 days). By 90 days (3 months), 62.9% of mt-sDNA users had completed FU-CY, compared to only 22.8% of FIT-FOBT users, reflecting a marked difference in early adherence rates. By 180 days (6 months), adherence among mt-sDNA users increased to 78.6%, representing 92% of their total adherence by 12 months, which ultimately reached 85.0%. In contrast, FIT-FOBT users showed significantly slower and lower adherence throughout the year, with only 29.8% completing FU-CY by 6 months and just 35.2% by 12 months. This aligns with findings from a previous study,^49^ which demonstrated that 90% of FU-CY adherence at 12 months can be achieved by 6 months. Given the critical importance of early detection, these findings underscore the need to encourage adherence within the earliest feasible follow-up periods.

While the FU-CY rate following a positive mt-sDNA reported here is impressive, it is possible that the rate could have been even higher were it not for the well-documented backlog of colonoscopy procedures.^50^, ^51^, ^52^, ^53^ This backlog has been attributed to both the COVID-19 pandemic and the dramatic overuse of colonoscopy for screening and other preventive indications.^50^, ^51^, ^52^^,^^54^ Given the recent and continued growth in the use of mt-sDNA testing,^55^ the need for FU-CY will only increase, and therefore fewer screening colonoscopies may need to be performed to accomodate more FU-CY procedures after a positive mt-SDNA test.

## Limitations

This study acknowledges several limitations that may affect the interpretation of its findings. First, adherence was evaluated over a 1-year period, which does not capture potential variations in follow-up over longer durations. Second, the change in guidance from the US Preventive Services Task Force lowering the recommended screening age from 50 to 45 occurred during the study period. This shift may have impacted the results by introducing additional heterogeneity in the population seeking out SBTs before and after the guidance change. Third, approximately 80% of mt-sDNA users in the study had commercial insurance, which may introduce selection bias. It is possible that individuals with commercial insurance may have better access to healthcare resources and follow-up services, potentially inflating adherence rates relative to other payer groups. The high proportion of commercially insured patients could have influenced our results, particularly since cost considerations often affect screening method selection.^56^ This relationship between adherence rates and cost-effectiveness is particularly important when evaluating the overall economic value of different CRC screening strategies in diverse patient populations.^57^

The results of this study have several implications for both clinical care and community-based CRC screening initiatives. Given that FU-CY adherence rates were significantly higher for mt-sDNA compared to FIT-FOBT in this age group, these findings highlight the potential advantages of mt-sDNA in promoting more timely and effective follow-up among younger adults. This is particularly relevant as early-onset CRC continues to rise, and timely FU-CY after a positive SBT is critical for early detection and intervention. From a clinical care perspective, these results emphasize the need to prioritize strategies that promote adherence to FU-CY, especially among populations newly eligible for screening. Tailoring outreach and follow-up protocols to optimize adherence within the earliest feasible timeframe is critical, particularly in younger populations, where early detection can significantly improve outcomes. In community-based settings, these findings emphasize the need to address barriers to FU-CY adherence, such as limited access to care and provider follow-up, to ensure more equitable outcomes. By addressing barriers to FU-CY adherence and leveraging the strengths of mt-sDNA, CRC screening programs can more effectively reduce the burden of early-onset CRC.

## Conclusions

In this retrospective study, FU-CY adherence after a positive SBT was significantly higher for mt-sDNA (85.0%) compared to FIT/FOBT (35.2%) among individuals aged 45–49. Among patients who completed a FU-CY, the mean time to FU-CY was observed to be shorter with mt-sDNA (74.5 days; median, 53 days) compared to FIT/FOBT (90.8 days; median, 63 days). These findings suggest that mt-sDNA is a promising tool to improve FU-CY adherence rates and enhance the quality of CRC screening in younger patient populations. By promoting earlier and more consistent adherence, mt-sDNA has the potential to play a pivotal role in optimizing CRC screening programs for younger, average-risk individuals, where timely detection can significantly improve outcomes.
